# Changes in Pharyngeal Airway Space and Oxygen Saturation Following Mandibular Setback Surgery: A Narrative Review

**DOI:** 10.7759/cureus.31178

**Published:** 2022-11-06

**Authors:** Bader Fatani, Omar Fatani, Ahmed Fatani, Jumana A Fatani, Afraa Al-Safadi

**Affiliations:** 1 Dentistry, King Saud University, Riyadh, SAU; 2 Medicine, King Saud University Medical City, Riyadh, SAU; 3 General Dentistry, King Saud University, Riyadh, SAU; 4 Surgery, Specialized Medical Center, Riyadh, SAU; 5 Pharmacy, King Khaled University Hospital, King Saud University Medical City, Riyadh, SAU

**Keywords:** pharyngeal, sleep apnea, oxygen saturation, airway space, mandibular setback

## Abstract

Orthognathic surgery can alter the dental, skeletal, soft tissue, and dimensional changes in the oropharyngeal region. Studies have shown a posterior positioning of the hyoid bone and tongue position after mandibular setback surgery, this in turn can have a negative influence on the upper airway space that could lead to breathing problems such as obstructive sleep apnea. Mandibular setback surgery is commonly used for functional and aesthetic correction in mandibular prognathism patients. There are still some controversial opinions regarding the effect of mandibular setback surgery on the pharyngeal airway space and oxygen saturation. This study aims to review and illustrate the changes in pharyngeal airway space and oxygen saturation following mandibular setback surgery.

## Introduction and background

Angle class III malocclusion prevalence differs between different population groups and ranges from 0% to 10% [1]. Asian population demonstrates the highest prevalence of angle class III malocclusion [1,2]. These days, the treatment of class III patients using bimaxillary surgery is increasing [3]. Yet, mandibular setback surgery is still the first approach in a lot of cases with prognathic mandibles [3,4]. Orthognathic and orthodontics treatment is unavoidable in adult patients with class III with an excessive negative overjet to ensure improved aesthetics, orofacial harmony, and mastication in these patients [1]. Orthognathic surgery can alter the dental, skeletal, soft tissue, and dimensional changes in the oropharyngeal region. Previous studies showed a posterior positioning of the hyoid bone and tongue after mandibular setback surgery, this, in turn, can have a negative influence on the upper airway space that could lead to breathing problems such as obstructive sleep apnea [1,2]. This study aims to review and illustrate the changes in pharyngeal airway space and oxygen saturation following mandibular setback surgery.

## Review

Methods

This study involved a review of published articles discussing the changes in the pharyngeal airway space and oxygen saturation following mandibular setback surgery. Several databases including PubMed, Web of Science, and Google Scholar were used to gather the most relevant studies. A search set was applied to combine a range of keywords: (Mandibular setback), (Pharyngeal airway) and (Oxygen saturation). By using this method, all the articles discussing the changes in pharyngeal airway space and oxygen saturation following mandibular setback surgery were obtained. In inclusion criteria, we included all the relevant studies discussing the effect of postoperative changes in the pharyngeal airway and oxygen saturation after mandibular setback surgery. The studies that had insufficient data, poor methodological quality, or outdated studies were excluded. The initial screening revealed 98 studies related to the changes in pharyngeal airway space and oxygen saturation following mandibular setback surgery. After applying our selection criteria, the most relevant studies were chosen and used in this current review. This study was conducted by reviewing 26 articles related to the postoperative changes in the pharyngeal airway and oxygen saturation after mandibular setback surgery.

Mandibular setback surgery

**Figure 1:**
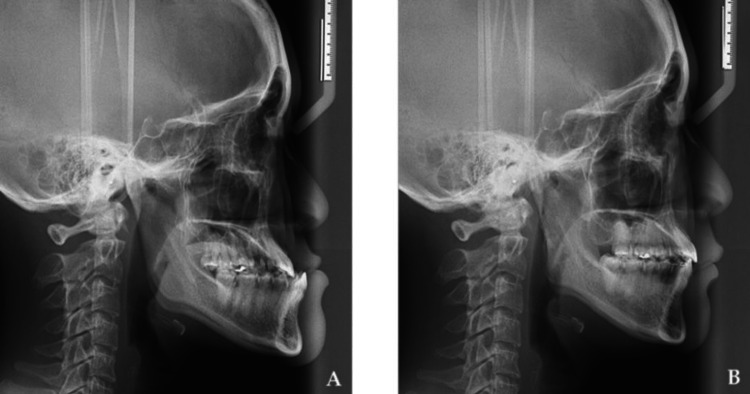
Demonstration of the changes in the pharyngeal airway space following mandibular setback surgery: (A) preoperative cephalogram and (B) post-operative cephalogram. Copyright: © 2021 by the authors. Licensee MDPI, Basel, Switzerland. This article is an open access article distributed under the terms and conditions of the Creative Commons Attribution (CC BY) license (https:// creativecommons.org/licenses/by/ 4.0/)  [7].

In orthognathic surgery, the mandibular setback is commonly performed using the bilateral sagittal split osteotomy to improve aesthetics, masticatory function, and occlusion through changes in the mandibular position [5]. Mandibular setback surgery is commonly used for functional and aesthetic correction in mandibular prognathism patients [6,7]. Mandibular prognathism and anterior open bite commonly present with higher gonial angle, macroglossia, forward tongue position, and higher mandibular plane angle. Physiological adaptation in patients with anterior open bite often includes anterior tongue placement. In addition, patients with anterior open bite commonly have inferiorly positioned hyoid bones and more constricted upper airways. When a posterior surgical setback of the mandible is planned, a partial glossectomy is often needed. Patients with anterior open bite have a higher prevalence of tongue-to-oral space volume, thus it is logical to consider that the pharyngeal airway space can constrict after the mandibular setback of the enlarged and anteriorly positioned tongue after the closure of anterior open bite compared to patients without anterior open bite [6]. Demonstration of the changes in the pharyngeal airway space following mandibular setback surgery in Figures 1A, 1B.

Pharyngeal airway space

**Figure 2:**
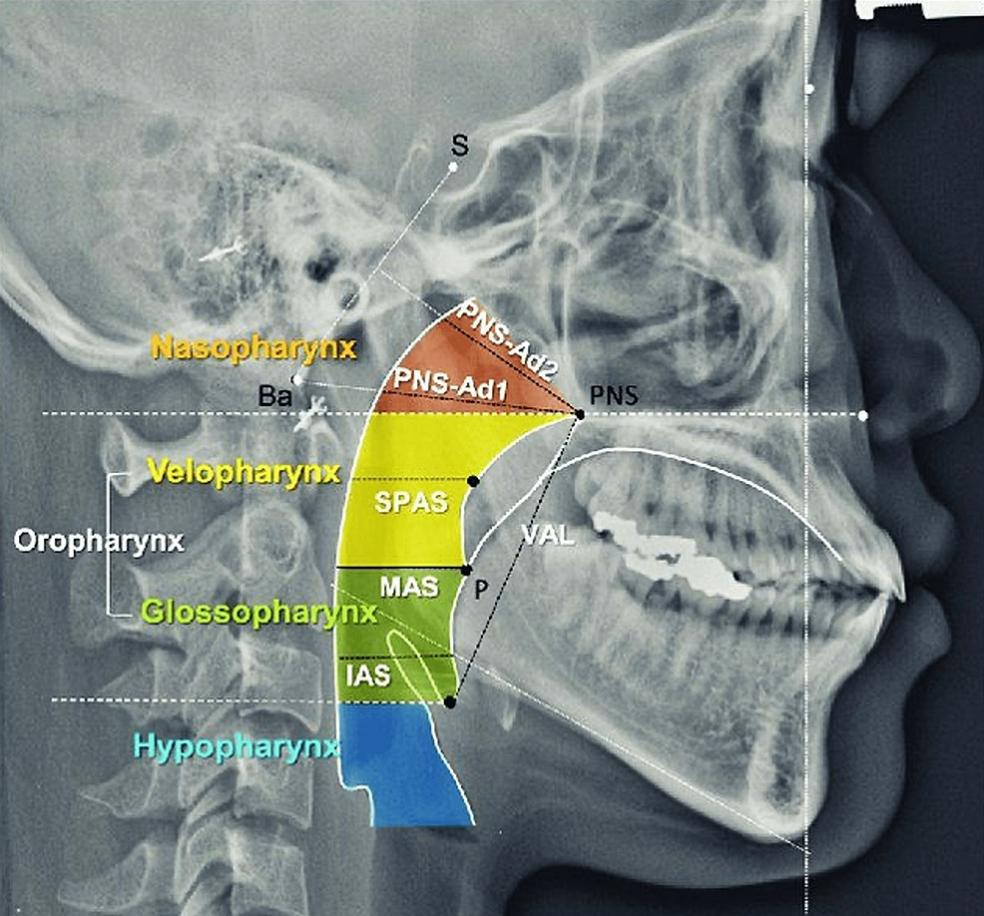
Demonstration of cephalometric pharyngeal evaluation to illustrate each pharyngeal airway section. © 2021 The Korean Association of Orthodontists. This is an Open Access article distributed under the terms of the Creative Commons Attribution Non-Commercial License (http://creativecommons.org/licenses/by-nc/4.0) which permits unrestricted non-commercial use, distribution, and reproduction in any medium, provided the original work is properly cited [12].

The pharynx is a tubular-shaped structure that travels from the base of the cranium to the sixth cervical spine. From inferior to superior, the pharynx is divided into three segments: the laryngopharynx, oropharynx, and nasopharynx. The midsagittal plane shows that the oropharynx and nasopharynx are separated by the hard palate. The laryngopharynx and oropharynx are separated by the epiglottis. The oropharynx is positioned posterior to the oral and nasal cavity and above the esophagus, trachea, and larynx. The oropharynx is divided into the retroglossal and retropalatal pharynx and separated by the soft palate. The tongue is the highest functioning part of the oropharyngeal structure and is affected by any alteration to the oro-dental system, particularly the mandible [2]. The changes in the position of hyoid bone are dependent on the resistance that is provided by the elastic membranes of the larynx and trachea and the action of supra-hyoid and infra-hyoid muscles [8]. The attached musculature and hyoid bone move backward and downward during mandibular setback surgery which causes changes in the supra and infrahyoid muscle tension [1]. This result in posterior positioning of the tongue and an increase in height which can affect the pharyngeal airway space, thus, careful estimation of tongue size is critical in diagnosis and treatment after mandibular setback surgery [1,2,9]. Genioplasty, debulking of the tongue, and bi-jaw surgery are considered alternative treatment options to prevent these unfavorable effects [1]. A previous study by Chen et al. showed that the pharyngeal airway space is negatively affected after mandibular setback surgery after the long-term follow-up [2]. A previous study done by Kang et al. demonstrated that mandibular setback surgery showed a significant reduction in pharyngeal airway compared to bimaxillary surgery (posterior impaction). In this study, bimaxillary surgery was further stable in terms of the airway. Thus, it is essential to assess the airway before surgery [9]. Fernández et al. reported that after mandibular setback surgery only, a significant decrease in area in the upper airway can persist in the long and medium term [10]. Kori et al. recommended bimaxillary surgery over mandibular setback surgery whenever possible to ensure that the hyoid bone is placed more posteriorly and inferiorly [8]. Irani et al. demonstrated that there is a major reduction in all pharyngeal airway volumes as well as anteroposterior and lateral surface dimensions at oropharyngeal up to one year after isolated mandibular setback surgery [11]. Demonstration of cephalometric pharyngeal evaluation to illustrate each pharyngeal airway section in Figure 2.

Oxygen saturation

The previous study done by a different group showed a significant reduction in oxygen saturation immediately following mandibular surgery that can spontaneously correct itself after one month [13]. Fernández et al. reported that the findings for arterial oxygen saturation postoperatively showed that the hypopnea, CT90 index, and O_2_ desaturation demonstrate no changes in long-term ventilation. Therefore, there is no clear evidence to confirm that mandibular orthognathic surgery leads to obstructive sleep apnea [10]. Pulse oximetry is commonly used for screening obstructive sleep apnea syndrome because changes in arterial oxygen saturation distinctly reflect hypopnea or apnea [14,15]. Mandibular advancement showed significant improvement in the oxygen desaturation index and respiratory disturbance index [13].

Sleep apnea

**Figure 3:**
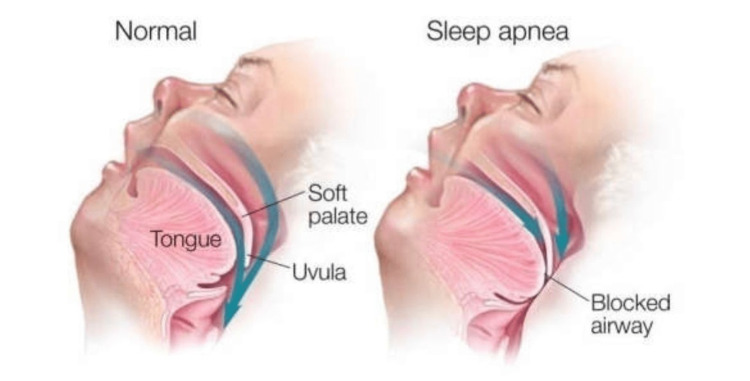
Demonstration of the normal airway and obstructive apnea. Copyright © 2019 Akhil K Padmanabhan et al. This is an open access article distributed under the Creative Commons Attribution License, which permits unrestricted use, distribution, and reproduction in any medium, provided the original work is properly cited  [21].

Obstructive sleep apnea is a type of sleep disorder commonly caused by airway collapse at several parts of the upper airway system which results in the obstruction of the airway [3,5]. It typically affects an average of 2%-4% of the adult population [16]. The chief concern with the changes in the pharyngeal dimensional is that there is a potential cause of obstructive sleep apnea [17]. However, a systemic review established that there is no strong relation between obstructive sleep apnea and mandibular setback surgery [18]. Obstructive sleep apnea has negative effects on general health and sleeps quality owing to continuous sleepiness throughout the day [2]. Many studies reported postoperative obstructive sleep apnea following mandibular setback surgery [9]. A previous study reported two cases of obstructive sleep apnea after mandibular setback surgery to treat prognathism of the mandible. Thus, maxillofacial surgeons should be careful during treatment planning for a significant amount of setback surgery to treat mandibular prognathism [2]. Previous studies reported that the incidence of mild to moderate obstructive sleep apnea syndrome is higher in mandibular setback surgery of 5 mm or more compared to that with less than 5 mm [3]. Lately, maxillomandibular advancement surgery is being considered a treatment choice to treat severe obstructive sleep apnea. Maxillomandibular advancement surgery enlarges anteroposterior and transverse airway dimensions, thus improving upper airway patency [19]. In case of increased airway resistance, apnea (absent airflow) or hypopnea (diminished airflow of at least 30%, lasting at least 10 seconds) could also occur [5,11]. A previous study also stated that some patients developed postoperative obstructive sleep apnea. In addition, the apnea-hypopnea index was slightly increased after the mandibular setback surgery [20]. Demonstration of the normal airway and obstructive apnea in Figure 3.

Diagnosis and treatment planning 

Surgeons should recognize the postoperative variations that follow mandibular setback surgery. In the past, cephalometric analysis was used to assess the results of a mandibular setback in the pharyngeal airway space. This method was useful to evaluate airway dimensions on the sagittal plane. However, it did not offer a view of the upper airway [3]. One of the limitations of lateral cephalogram is that it is a two-dimensional image and does not sufficiently represent a three-dimensional image [11,22]. These days, cone-beam computed tomography is reported to be useful in the diagnosis and analysis of surrounding airway space, soft tissue, and airway dimensions [3,11]. Yet, the American Association of Orthodontists stated that CBCT does not offer information regarding the actual function of the airway or neuromuscular tone [6]. Computed tomography and magnetic resonance imaging are the main two techniques used to diagnose and obtain 3D anatomical data to assess the changes in the posterior airway space changes [22,23]. The most accurate method to diagnose obstructive sleep apnea induced by surgery is overnight polysomnography (PSG) [17]. Overnight PSG and recording of the respiratory variables, including levels of oxygen saturation and respiratory disturbance index is the gold standard for diagnosing and treatment planning of obstructive sleep apnea before and after the surgery [16]. Computational fluid dynamics can be used as an airflow simulation and is recently used for patients with obstructive sleep apnea who was treated with genioglossal advancement, adenotonsillectomy, maxillomandibular advancement, or mandibular advancement devices [5]. The diagnosis of obstructive sleep apnea is five or more events of hypopnea or apnea per hour of sleep for at least 10 seconds [15,24]. Obstructive sleep apnea is usually associated with cardiovascular and cerebrovascular disease, excessive daytime sleepiness, and fatigue [5]. Positive airway pressure treatment is suggested for patients with severe obstructive sleep apnea. Yet, maxillomandibular advancement is advocated to increase the airway space in case patients with severe obstructive sleep apnea did not tolerate the therapy [20]. Surgeons should consider the possibility of obstructive sleep apnea in patients with obesity, snoring, large tongue, large mandibular setback, short neck, and excessive daytime sleepiness [5]. Surgeons should also avoid increasing the risk of developing obstructive sleep apnea by predicting the estimated reduction of the airway change after mandibular setback surgery [25].

Complications and risk factors

A previous study by Du et al. aimed to evaluate changes in the pharyngeal airway space in high body mass index patients, the results showed that in adults with high body mass index, mandibular setback (bilateral sagittal split ramus osteotomy) can reduce the pharyngeal airway space. On other hand, genioplasty advancement can enlarge the pharyngeal airway space after surgery. Thus, genioplasty advancement alongside mandibular setback (bilateral sagittal split ramus osteotomy) can help in reducing the adverse effects of a pharyngeal airway space reduction [26]. Lee et al. demonstrated that the pharyngeal airway space is narrower in patients with increased BMI, especially after surgery. Therefore, weight control is needed especially after mandibular setback surgery. Age factors can strongly be associated with the development of obstructive sleep apnea. The elderly patient shows changes in sleep patterns and a reduction in the neuromuscular tone including the genioglossus tone [19]. Obstructive sleep apnea is suggested to have a risk for cardiac arrhythmias, as well as systemic and pulmonary hypertension. Therefore, the frequency of using isolated bilateral sagittal split osteotomy has decreased to 10% and bi-jaw surgery increased to 40% in the treatment of class III skeletal deformities [8]. Obstructive sleep apnea is usually associated with cardiovascular and cerebrovascular disease, excessive daytime sleepiness, fatigue, sleep fragmentation, and metabolic disturbances, thus it is considered potentially life-threatening [5,6,16,25].

## Conclusions

Mandibular setback surgery is commonly used for functional and aesthetic correction in mandibular prognathism. Our study showed that mandibular setback surgery could affect the postoperative pharyngeal airway space as well as oxygen saturation, thus the clinician should recognize the postoperative variations that follow mandibular setback surgery. Moreover, the surgeon's main responsibility is to provide an adequate treatment outcome after the mandibular setback surgery while also considering all the postoperative complications.

## References

[1] Sahoo NK, Agarwal SS, Datana S, Bhandari SK (2021). Effect of mandibular setback surgery on tongue length and height and its correlation with upper airway dimensions. J Maxillofac Oral Surg.

[2] Chen KJ, Chen YT, Hsiao SY, Chen MY (2021). Postoperative changes in tongue area and pharyngeal airway space following mandibular setback surgery through intraoral vertical ramus osteotomy. Biomed Res Int.

[3] Kim SH, Choi SK (2020). Changes in the hyoid bone, tongue, and oropharyngeal airway space after mandibular setback surgery evaluated by cone-beam computed tomography. Maxillofac Plast Reconstr Surg.

[4] Tselnik Tselnik, M M, Pogrel MA (2000). Assessment of the pharyngeal airway space after mandibular setback surgery. J Oral Maxillofacial Surg.

[5] Yajima Y, Oshima M, Iwai T, Kitajima H, Omura S, Tohnai I (2017). Computational fluid dynamics study of the pharyngeal airway space before and after mandibular setback surgery in patients with mandibular prognathism. Int J Oral Maxillofac Surg.

[6] Lee K, Hwang SJ (2019). Change of the upper airway after mandibular setback surgery in patients with mandibular prognathism and anterior open bite. Maxillofac Plast Reconstr Surg.

[7] Chen CM, Yu TY, Chou ST, Cheng JH, Chen SC, Pan CY, Tseng YC (2021). Changes in tongue area, pharyngeal area, and pharyngeal airway velocity after correction of mandibular prognathism. J Clin Med.

[8] Kori C, Shetty P, Shetty M, Ravi MS (2022). Comparative evaluation of the effects of bimaxillary and mandibular setback surgery on pharyngeal airway space and hyoid bone position in skeletal class III patients. J Clin Exp Dent.

[9] Kang NE, Lee DH, In Seo J, Lee JK, Song SI (2021). Postoperative changes in the pharyngeal airway space through computed tomography evaluation after mandibular setback surgery in skeletal class III patients: 1-year follow-up. Maxillofac Plast Reconstr Surg.

[10] Fernández-Ferrer L, Montiel-Company JM, Pinho T, Almerich-Silla JM, Bellot-Arcís C (2015). Effects of mandibular setback surgery on upper airway dimensions and their influence on obstructive sleep apnoea - a systematic review. J Craniomaxillofac Surg.

[11] Irani SK, Oliver DR, Movahed R, Kim YI, Thiesen G, Kim KB (2018). Pharyngeal airway evaluation after isolated mandibular setback surgery using cone-beam computed tomography. Am J Orthod Dentofacial Orthop.

[12] Cho HN, Yoon HJ, Park JH, Park YG, Kim SJ (2021). Effect of extraction treatment on upper airway dimensions in patients with bimaxillary skeletal protrusion relative to their vertical skeletal pattern. Korean J Orthod.

[13] Foltán R, Hoffmannová J, Pavlíková G (2011). The influence of orthognathic surgery on ventilation during sleep. Int J Oral Maxillofac Surg.

[14] Kobayashi T, Funayama A, Hasebe D, Kato Y, Yoshizawa M, Saito C (2013). Changes in overnight arterial oxygen saturation after mandibular setback. Br J Oral Maxillofac Surg.

[15] Kitagawara K, Kobayashi T, Goto H, Yokobayashi T, Kitamura N, Saito C (2008). Effects of mandibular setback surgery on oropharyngeal airway and arterial oxygen saturation. Int J Oral Maxillofac Surg.

[16] Canellas JV, Barros HL, Medeiros PJ, Ritto FG (2016). Sleep-disordered breathing following mandibular setback: a systematic review of the literature. Sleep Breath.

[17] Yang HJ, Jung YE, Kwon IJ, Lee JY, Hwang SJ (2020). Airway changes and prevalence of obstructive sleep apnoea after bimaxillary orthognathic surgery with large mandibular setback. Int J Oral Maxillofac Surg.

[18] Jang SI, Ahn J, Paeng JY, Hong J (2018). Three-dimensional analysis of changes in airway space after bimaxillary orthognathic surgery with maxillomandibular setback and their association with obstructive sleep apnea. Maxillofac Plast Reconstr Surg.

[19] Lee ST, Park JH, Kwon TG (2019). Influence of mandibular setback surgery on three-dimensional pharyngeal airway changes. Int J Oral Maxillofac Surg.

[20] Kim JW, Kwon TG (2020). Why most patients do not exhibit obstructive sleep apnea after mandibular setback surgery?. Maxillofac Plast Reconstr Surg.

[21] K Padmanabhan, Akhil Akhil, Gautam Gautam (2019). Obstructive sleep apnea and oral health: a short review. Int J Curr Med Pharm Res.

[22] Uesugi T, Kobayashi T, Hasebe D, Tanaka R, Ike M, Saito C (2014). Effects of orthognathic surgery on pharyngeal airway and respiratory function during sleep in patients with mandibular prognathism. Int J Oral Maxillofac Surg.

[23] Karan NB, Kahraman S (2019). Evaluation of posterior airway space after setback surgery by simulation. Med Biol Eng Comput.

[24] Hasebe D, Kobayashi T, Hasegawa M (2011). Changes in oropharyngeal airway and respiratory function during sleep after orthognathic surgery in patients with mandibular prognathism. Int J Oral Maxillofac Surg.

[25] Chen F, Terada K, Hanada K, Saito I (2005). Predicting the pharyngeal airway space after mandibular setback surgery. J Oral Maxillofac Surg.

[26] Du W, He D, Wang Y, Liu H, Liao C, Fei W, Luo E (2017). Upper airway changes after mandibular setback and/or advancement genioplasty in obese patients. J Oral Maxillofac Surg.

